# Mycorrhizas and Trichoderma fungi increase the accumulation of secondary metabolites in grain legume leaves and suppress foliar diseases in field-grown conditions of the humid forest of Cameroon

**DOI:** 10.1186/s12870-023-04587-z

**Published:** 2023-11-21

**Authors:** Martin Jemo, Severin Nkenmegne, Alfred Balenor Buernor, Anas Raklami, Zachee Ambang, Adamou Souleyamanou, Yedir Ouhdouch, Mohamed Hafidi

**Affiliations:** 1 AgroBiosciences Program, College of Agriculture and Environmental Sciences (CAES), Mohammed VI Polytechnic University (UM6P), Lot 660, Hay Moulay Rachid, Benguerir, 43150 Morocco; 2 Department of Plant Biology, Faculty of Science, University of Yaoundé I, P.O. Box. 812, Yaoundé, Cameroon; 3 Laboratory of Microbial Biotechnologies, Agrosciences and Environment, (BioMAgE) Labeled Research Unit-CNRST N°4, Faculty of Sciences Semlalia, University Cadi Ayyad (UCA), Marrakech, BP 2390, Morocco; 4 African Sustainable Agriculture Research Institute (ASARI), Mohammed VI Polytechnic University (UM6P), Laayoune, 7000 Morocco

**Keywords:** Common bean, Mycorrhiza symbiosis, Field assessment, Low molecules weight, Soybean

## Abstract

**Background:**

Arbuscular mycorrhizal and Trichoderma fungi alter the synthesis of secondary metabolites of plants and confer tolerance from pathogens attacks. However, there is less supportive evidence from on-field studies confirming the above-mentioned hypothesis, particularly for the humid forest zone of Cameroon where pathogens are important sources of yield losses for legumes such as soybean and common bean.

**Materials and methods:**

We evaluated the impacts of mycorrhiza isolates of *Rhizophagus intraradices* (Ri) and *Trichoderma asperellum* (Ta) fungi and their co-inoculations (Ta x Ri) in the synthetizing of leaves secondary metabolites, foliar disease symptoms, growth, N and P uptake, and yields of three genotypes of soybean (TGx 1485-1D, TGx 1990-93 F, and TGx 1990-97 F) and common beans (NUA-99, DOR-701, and PNN) under field conditions of Cameroon.

**Results:**

We found that common bean plants showed a lower foliar infection rate but a higher increase in root colonization intensity, shoot dry weight, and N and P uptakes than soybeans when inoculated with Ri and Ta treatment. However, the grain yield of soybean soybean was higher (2000 kg ha ^1^) than the common bean plants for the Ri × Ta treatment. The soybean genotype TGx 1990-93F had increased root colonization intensity and the lowest foliar infection rate, making it stronger and tolerant to pathogen attacks when co-inoculated with Ri × Ta fungi (F). Bean plants inoculated with Ri and the co-inoculated with Ri × Ta demonstrated lower symptoms of foliar attack, and increased root colonization, particularly the PNN variety. The total amino acid and proline accumulations were higher for soybean than common bean plants due to fungi inoculations, and soybean genotypes accumulated more excellent contents of amino acid and proline in the control (10.1 mg g^− 1^ fwt) that significantly increased under the Ri × Ta inoculation (13.4 mg g^− 1^ fwt).

**Conclusions:**

Common bean plants inoculated with Ta and Ri fungi accumulated higher phenolic compounds in their leaves that aided them in overcoming the pathogen attacks than soybean plants.

**Supplementary Information:**

The online version contains supplementary material available at 10.1186/s12870-023-04587-z.

## Background

Grain legumes like soybean (*Glycine max* L) and Common bean (*Phaseolus vulgaris* L.) play essential roles in the sustainability of agricultural systems and contribute to the food and nutrition security of people, especially in Africa [1]. In many production systems, these crops are valued in rotations or intercropping for their ability to transform atmospheric nitrogen (N_2_) into usable N forms, reducing their own and associated/intercropped non-legume N needs due to an intimate symbiotic relationship with bacteria, known as “rhizobia” [2, 3]. Another advantage of legume cultivation is its rich protein contents in grain that can range from 20 to 40%, depending on the species and variety [4]. However, despite contributions mentioned above of legumes to quality nutrition and food security maintenance, the production is severely compromised by diverse root and foliar diseases caused by necrotrophic pathogens, resulting in low yields, particularly in smallholder farms in Africa [5, 6]. Other impediments to higher grain legume yields are the divergent abiotic factors, such as the low nutrient availability of soils [7].

To prevent losses from pest and disease attacks, growers need to apply pesticides that require several applications of inorganic chemical molecules to increase yield but also have adverse effects on humans and the environment. Furthermore, the market’s ever-increasing demands for more evergreen and healthy food products free of chemicals, coupled with legislative pressures for reducing the reliance on pesticides, impose alternative production methods [8–10]. There is an urgent need to develop alternative production systems, such as using biocontrol antagonistic or plant growth-promoting microbes capable of activating systemic defense mechanisms of plants against a broad spectrum of pathogens and mimicking their incidence [5, 11]. For instance, the well-known soil inhabiting arbuscular mycorrhizal fungi (AMF) and Trichoderma species have been widely reported for their success in sustainable farming towards a wide range of host plants [12]. However, there is a dearth of evidence pinpointing the plant-protecting abilities and ensuring yield increase from inoculations with mycorrhizal and Trichoderma fungi for diverse grain legumes (i.e., soybean, common bean) in the humid forest zone of Cameroon, particularly for studies performed under field conditions [12].

Trichoderma is a cosmopolitan and opportunistic ascomycete fungal genus with several species of interest to agriculture as direct biocontrol agents of phytopathogens and plant growth promotors [9, 11]. The fungi utilize direct antagonism and competition mechanisms, particularly in the rhizosphere, to modulate and alter the composition of other cohabiting microorganism [9]. Penetrations into the roots by Trichoderma mycelia induce substantial changes in the host plants’ metabolism and activate a systemic defense response that is effective against a broad spectrum of pathogens [13]. In vivo culture of *T. asperellum* strain produces antibiotic metabolites to inhibit pathogen fungi through antibiosis [5]. Furthermore, Trichoderma strains synthesize the *β* I-3 glucanases, chitinases, and proteinases molecules to parasitize the pathogen’s hyphae and sclerotia, invading the cells and causing lysis [5]. AMF constitutes a dominant component of soil-living organisms that symbiotically associate with roots [14]. Various AMF species are resident of the rhizosphere, with a wide array of species forming mutualistic associations with plant roots [10]. Upon root colonization, AMF develops intra-tree-like structures, “Arbuscules”, extra mycelia structures to access nutrients such as phosphorus (P) to promote host plant growth, and improve stress resistance [15, 16]. Like Trichoderma, AMF can alter the physiology of the host plant by producing an extensive range of structurally varied low molecular weight secondary metabolites such as volatile organic compounds [17] and phenolic compounds [18] and activate the critical defense-related enzymes from hosts [10] to confer protection against pathogen attacks. In the context of farming systems in the humid forest zones of Cameroon, both AMF and Trichoderma fungi are being regarded as consortia of biocontrol agents to promote plant growth and reduce pathogens and pest attacks, owing to a plausible synergistic relationship from the two mutualists microorganisms [12]. Nonetheless, a proper understanding of the interactions between AMF and Trichoderma species is still incomplete. There are no conclusive results from field studies about foliar disease suppression for many grain legumes, such as soybean and common bean.

The use of AMF and *Trichoderma* as biocontrol agents, either inoculated separately or in consortia inocula, to control pathogens and pest attacks and foster growth promotion of legume plants have been the subject of studies under greenhouse conditions in Cameroon [12]. Using co-inoculation with a Trichoderma strain and AMF inocula, the authors observed that the P uptake of beans increased compared to single-inoculated plants. Moreover, the disease incidence and severity were reduced upon the Trichoderma strain and AMF inocula combination. Abdelmoteleb et al., [13] evaluated cell-free culture filtrate of *T. longibrachiatum* to control *Fusarium solani* and induce a defense response in *P. vulgaris* L. plants. The results showed that the cell-free culture filtrate of Trichoderma at different concentrations of isolated *T. longibrachiatum* inhibited mycelial growth of *F. solani* with growth reduction ranging from 28.8 to 97.3% and inhibited spore germination up to 96% compared to the control treatment and upregulation of defense-related genes. However, few studies on the antagonistic potential of Trichoderma species to eliminate antagonistic fungi and adversely impact other beneficial fungi could also compromise the cooperation between between AMF and Trichoderma species, and therefore require integrating synergisms or compatibilities between the fungi for optimal benefits in the product development [5]. Inoculation of mycorrhizal plants with *T. aureoviride* produced more biomass, while there was no effect in non-mycorrhizal plants, indicating a synergistic interaction that can be exploited for the development of consortia biocontrol agents. Benefiting from the synergetic effects resulting from the co-inoculation of AMF and Trichoderma species to trigger plant defense and promote plant growth is advocated for application in modern agriculture.

In the humid areas of Cameroon, various soil-borne or foliar diseases, including, *Cercospora sojina, Phakopsora pachyrhizi, Fusarium graminearum, C. kukuchii, and Corynespora cassiicola, and P. pachyrhizi Sydow for soybean, and Pseudocercospora griseola, Uromyces appendiculatus, F. solani, F. oxysporum, and Colletotrichum lindemuthianum* for common bean are sources of huge yield losses [12, 19]. Among these diseases, dominant proportions are from fungal origins, and Asian rus caused by *P. pachirhizi* represents a major constraint for soybean production, causing up to 90% of yield losses [19]. With common bean, *P. griseola* responsible for angular leaf spots are responsible are responsible for over 80% of yield reduction of the bean cultivar in the humid highland of Cameroon [20]. The post-emergency aboveground symptoms involve loss of vigor, leaf yellowing lesions, leaf drop, severe defoliation, plant lethality, and yield losses to grain legumes [7–10]. The overall objective was to assess whether the consortium of AMF and Trichoderma spp. enhance the tolerance of soybean and common bean seedlings towards necrotic foliar diseases attack under frequent humid forest soil conditions of Cameroon. Specifically, we aimed to:

- Study the effects of AMF (*R. intraradices*) and Trichoderma *(T. asperellum*) fungi to colonize roots, reduce foliar disease severity, and promote the growth of soybean and common bean plants.
- Explore the genetic variation among soybean and common bean co-inoculated with *R. intraradices* and *T. asperellum* fungi in terms of disease severity reduction and growth improvement.
- Investigate the species difference between soybean and common bean and their intra-genotypic/varietal variation for the accumulation of secondary metabolites of inoculated plants with *R. intraradices, T. asperellum*, and co-inoculated with both fungi.

## Materials and methods

### Biological materials

Three soybean varieties acquired from the International Institute of Tropical Agriculture (IITA) in Nigeria were used to establish the field trial. The varieties derived from the tropical glycine max (TGx) series were TGx 1485-1D, TGx 1990-93F, and TGx 1990-97F. The variety TGx-1485-1D was released in 1990 and registered under national code: NGGM-96-13 and is from the extra early maturity group (https://www.seedportal.org.ng). Also, three common bean varieties, NUA-99, DOR-701, and PNN, released by the Institute de le Recherche Agriculture et Development of Cameroon, were used in the trials (https://www.pabra-africa.org/cameroon-releases-new-five-bean-varieties). The bean variety NUA-99, bred by the International Center for Tropical Agriculture (CIAT), is a large seed bean with a red and white tined color. The variety has a growth cycle from 70 to 75 days and a yield ranging from 1.5 to 2.5 tons ha^− 1^. The bean variety DOR-701 has a longer production cycle from 80 to 90 days and the seeds are bright red in color. The variety also originated from CIAT with a yield range between 2 and 3 -tons ha^− 1^. The variety PNN is a landrace with with black-colored seeds, growing cycles of about 90 days, and a yield range between 1.2 and 2.5 -tons ha^− 1^.

The mycorrhizal inoculant “Rhizatech” containing isolates of *R. intraradices* was obtained from Dudutech Company, Kenya (http://dudutech.com/products/rhizatech). The inoculant contained spores and colonized maize root fragments prepared with a granular carrier at a concentration of 50 propagules per cubic centimeter of the *R. intraradices* isolates.

The Trichoderma product “Trichotech” was obtained from Dudutech, Kenya, and contained spores of *T. asperellum*, an antagonistic fungus to control soil-borne fungal diseases including Fusarium spp., Rhizoctonia spp, Sclerotinia spp., and Pythium spp in plants. The product contains spores and mycelial fragments of *T. asperellum* Isolate H22 at a concentration of 4 × 109 CFUs per gram.

### Experimental site

The experiment was conducted at Nkolbisson (3°51’ N, 11°25’ E), 8 km West of Yaoundé at an altitude of 780 m above sea level (asl). The climate belongs to a tropical humid rainforest zone forest zone with a mean annual rainfall approximating 1,663 millimeters for the year 2013, and mean annual temperatures vary from 25 to 27 °C. The soil at the site is classified as Rhodic Kandiudult type, presenting a low exchange capacity, high anion absorptive capacity, with essentially red or yellowish color, and strong acidic reaction (USDA soils classification system). The natural vegetation in the site was dominated by Mucuna (*Mucuna sp*.), and the site was under one-year-old fallow. Cassava (*Manihot esculenta* L. Crantz) was the precedent crop harvested before the bush fallow establishment. The field site was manually cleared off from bush fallow and before the trial was established. The soil at the site had a low available P level [21].

### Soil sampling and analyses

Composite soil samples were sampled from 0 to 20 cm in four replicates and transferred to the laboratory for chemical analyses. The soil was air-dried and sieved to pass through 1-mm mesh, and the chemical properties were analyzed in 4 analytical replicates. The soil-pH was measured in aqueous soil suspension (1:2.5, v: v) using the Corning 125 pH meter (Corning Life Sciences, Amsterdam, Netherlands) after agitating the sample for 16 h. The soil available-P was determined by the Bray-I chemical extraction method. Briefly, 30 ml of Bray-I extractant was added to 3 g of the air-dried soil sample, and the content (soil solution ratio 1:10) was shaken for 5 min and filtered, and the P concentration was measured after the colorimetric change [22]. Total N concentration was measured in a subsample of 0.5 g after digestion with concentrated H_2_SO_4_ at 500 °C using a stainless-steel pressure digestion system (BERGHOF Products + Instruments GmbH Labor-Technik Eningen, Germany). Organic C was determined by chromic acid digestion and a spectrophotometric procedure [23]. The N concentration was determined using a spectrophotometer (Jenway 6310 Scanning Visible Range Spectrophotometer 230 V, Clarkson Laboratory, USA), according to the method described by Novozamsky et al. [24].

### Experimental set-up

The design of the field trial was a factorial with three replicates and the following factors tested for each legume: (a) factor 1, grain legume variety with three levels (three varieties for each), (b) factor 2: biocontrol application with four levels: [control, *T. asperellum* (Ta), inoculation with *R. intraradices* (Ri), and the Ri × Ta combination]. Each plot measured 4 × 4 m, and five seeds per hill were sown at 0.25 m within each row and 0.50 m between each row and thinned to three seedlings per hill after germinations to target a population density of 240,0000 plants per hectare. Soybean seeds were sown 0.05 m within and 0.75 m between lines, reaching a population density of 266,666 plants per hectare. Inoculation of soybean and bean plants with mycorrhiza was done by placing 25 g of inoculant into the seed hole and gently recovering with fine soils. For the Trichoderma treatments, three applications were conducted as follows: 125 g of Trichotech product was poured into 100 L of water and mix thoroughly and sprayed in the entire plot one week, three days, and a day before sowing. The subsequent applications were conducted a month after the seedling emergency, and the last at the flowering stage. The plots were hand-weeded, when necessary, until the harvesting of plants.

### Identification of foliar disease-causing agents and severity during growth development

During the growth stages of the soybean and bean plants, i.e., from seedlings to mid-pod fill (45–50 DAS), regular field visits were conducted, and observations on leaf yellowing lesions were collected from each plot. The leave symptoms observed and the detection of the fungi causing the associated disease were done using a commercial mobile application AGRIO (https://agrio.app/). Five leaves of three plants were scanned using the AGRIO application connected to a cloud, and the associated fungal disease was with 95% similarity proposed and recorded. The AGRIO application uses artificial intelligence and machine learning approaches to simulate each image and millions of similar images constructed in the cloud. We also manually assessed the damages from each leaf by scoring for the presence/absence of lesions on ten plants for yellowing lesions [25] The severity of leaf (SL) of yellowing lesions (SLL) was calculated following Eq. 1.

1 $$\begin{array}{l}{\rm{SL}}\,\left( {\rm{\% }} \right)\,{\rm{ = [}}\left( {{\rm{Total}}\,{\rm{of}}\,{\rm{plants}}\,{\rm{sampled}}} \right)\, - \,\left( {{\rm{Number}}\,{\rm{of}}\,{\rm{plants}}\,{\rm{with}}\,{\rm{yellowing}}\,{\rm{lesion}}\,{\rm{symptoms}}} \right)\\{\rm{/}}\left( {{\rm{Total}}\,{\rm{of}}\,{\rm{sampled}}\,{\rm{plants}}} \right){\rm{]}}\, \times \,{\rm{100}}\end{array}$$

At the mid-pod fill stage, three plants of soybean and bean from rows of each plot were cut at 5 cm above ground level, and the fresh biomass was recorded. Ten grams of fresh leave subsamples were kept fresh at 4 °C for biochemical analysis for the total soluble amino acid and proline contents. A representative subsample of 500 g was taken to the laboratory and oven-dried at 70 °C for 72 h for dry weight measurement and subsequently used for tissues N and P analysis. The roots were gently removed from the soils, washed carefully to remove the soils attached to the roots, macerated, and the fresh weight measured. A subsample of one gram of fresh root was chopped into one cm lengths and preserved in 70% ethanol for root length colonization by mycorrhizal analysis.

### Total N and P tissue analyses and root colonization rate

Subsamples of dry shoot matter were finely ground to pass through one-mm mesh, and 0.5 g was digested in concentrated H_2_SO_4_ at 500 °C. The N concentration was measured according to the Novozamsky et al. [24] method and the total P concentration was determined by the colorimetric method following the Murphy and Riley, [22] procedure. The roots were stained following the Phillips and Hayman [26] and Brundrett et al. [27] protocols. Briefly, the roots were poured off the ethanol solution, cut into fragments of about one-cm in length, and macerated in 10% (w/v) KOH at 80 °C for one h. Then, they were neutralized with 1% HCl for 20 min at room temperature and stained with a mixture of Trypan and Methylene Blue (0.05% each in lactoglycerol) at 80 °C for one h, and de-stained in water overnight. According to McGonigle et al. [28], root length colonized by mycorrhizal mycelium was scored at 200× magnification using an Olympus AX70 microscope.

### Secondary metabolites

The total soluble amino-acid and proline compounds were extracted from one-gram fresh soybean and bean plant leaves ground in a mortar with 10 ml alcohol ethanol pure solution 80% (alcohol-water solution 80 − 20(v/v). The total soluble amino-acid, proline concentrations were determined by the modified ninhydrin acid reaction method of Yemm et al. [29]. Succinctly 50 µL of the extract was added to 0.5 mL of 0.2 M citrate buffer pH 5, 1 mL alcohol solution, and 1 mL ninhydrin acid reaction (1% de ninhydrin acid and 0.006% KCN in acetone). The mixture was placed in heated water at 60 °C for 15 minutes and then cooled down immediately in an ice bath for 5 minutes. The absorbance of amino acid and proline were measured, respectively at 570 *ƞ*m and 440 *ƞ*m with a spectrophotometer (Jenway 6310 Scanning Visible Range Spectrophotometer 230 V, Clarkson Laboratory, USA).

The leaf phenolic content was determined using 1 mL of each sample, mixed with 1 ml of 95% ethanol, 4 mL of deionized water, 0.5 mL of Folin-Ciocalteu reagent, and 1 mL of 0.5% sodium carbonate. Mixtures were then placed in the dark for 60 min, and afterward, the absorbance rate was measured at 725 nm. Gallic acid was used as the standard solution, where the concentrations of soluble phenolic compounds were expressed as mg g^− 1^ fwt [30].

### Grain yield harvest

At grain maturity between 90 and 100 DAS, a subplot of 2 × 2 m (4 m^2^) was delimited from each plot, the number of plants recorded, and pods harvested. Grains were separated from pods and sun-dried to achieve about 12% relative humidity, and the dry weight of the seed was measured. The seed moisture content was adjusted to 12% of grain humidity expressed in (kg ha ^− 1^) and was computed using the following Eq. 2.

2 $$\begin{array}{l}{\rm{Yield}}\,{\rm{(kg/ha)}}\, = \,[({\rm{Net}}\,{\rm{plot}}\,{\rm{yield}}\,{\rm{(g)}}\,/1000\,{\rm{(g)}}\, \times \,(({\rm{Area}}\,{\rm{(ha)}}\,10000\,(m2)\,/\\{\rm{Net}}\,{\rm{plot}}\,{\rm{area}}\,{\rm{(m2)}}\, \times \,((100\, - {\rm{MC)}}/88)],\,{\rm{where}}\,{\rm{MC}}\,{\rm{is}}\,{\rm{moisture}}\,{\rm{content}}\,(\% )\end{array}$$

### Statistics

Statistical analyses of the data were carried out using the Statistical Analysis System software version 9.2 (2009). A one and two-way analysis of variance (ANOVA) was carried out to assess the individual effect of legume species, inoculation treatments, and their interactions using the JMP statistical software (JMP, 2019). The TUKEY’s test method was used to separate means that were different at *p* ≤ 0.05. Levels of significance are given by ‘ns’ (not significant, *p* > 0.05), *p < 0.05, **p < 0.01, and ***p < 0.001. Values in columns followed by the same letter are not significantly different at *p* < 0.05 (LS-means/PDIFF option).

## Results

### Chemical properties of soils at the field site

The average soil-pH value was 5.2 ± 0.2 (mean ± s.e), and the P concentration was 3.3 ± 0.3 mg kg^− 1^. Other chemical soil properties analyzed were as follows: organic carbon concentration 129.6 ± 26 mg C, and total nitrogen concentration 16.5 ± 4.5 g kg^− 1^ soil (Table S1).

### Identification of foliar disease-causing agents on soybean and bean

**Fig. 1:**
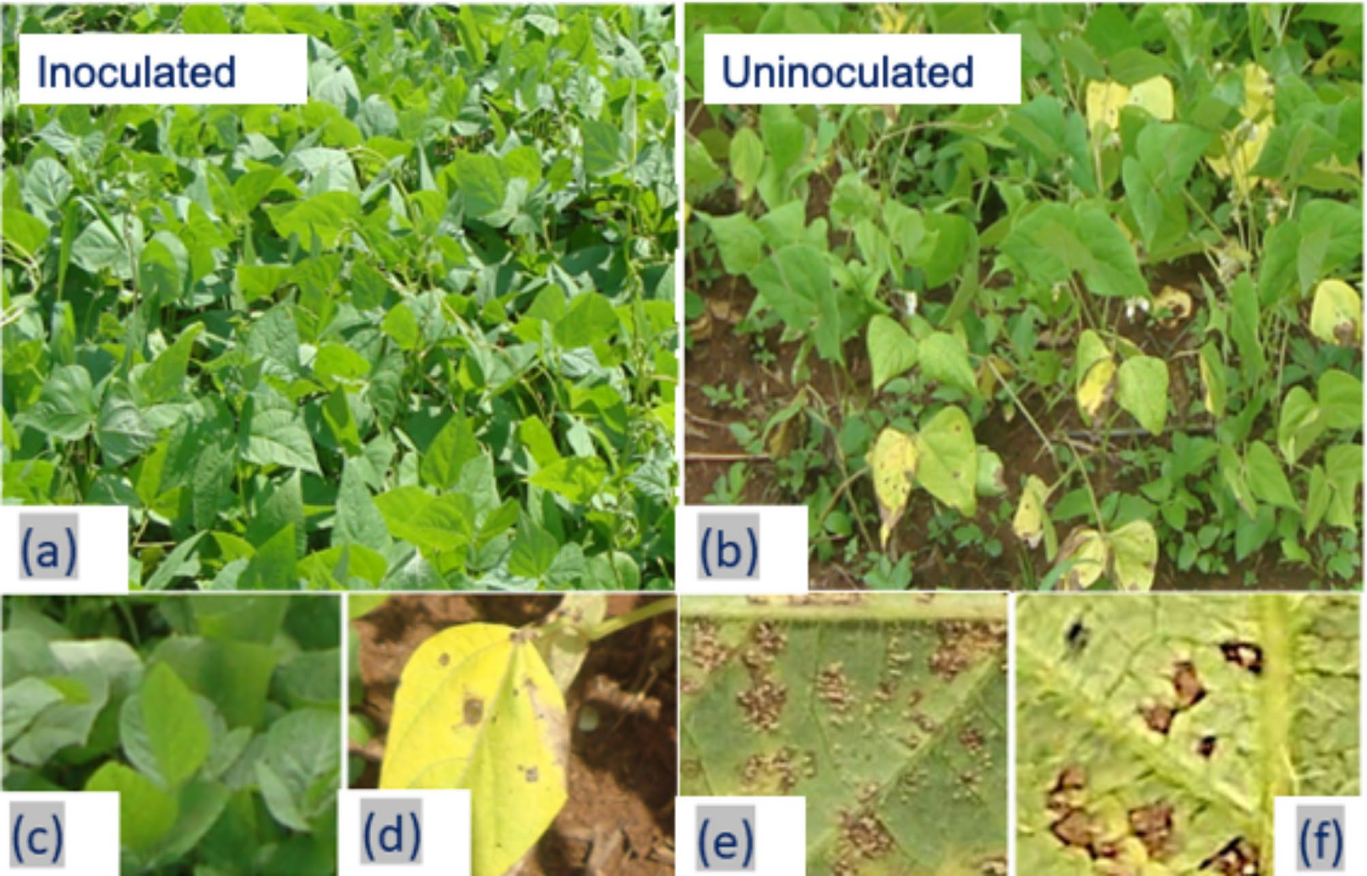
Inoculated (**a**) *Rhizophagus intraradices and Trichoderma asperellum* and uninoculated (**b**) soybean and bean plants showing symptoms of foliar diseases attack with the severity lesions and yellow color of leaves color. (**c**) magnified leaves of healthy soybean plants, (**d**) leaf spot, (**e, f**) leaf lesions of about 1 cm diameter caused by *Pseudocercospora griseola* on Common bean leaves

The AGRIO application allowed the identification of several causing agents of foliar diseases on the leaves of soybean and bean plants. Several causing agents were detected for soybean, principally, *C. sojina, P. pachyrhizi, F. graminearum, C. kukuchii, C. cassiicola, P. pachyrhizi Sydow*, and *P. pachirhizi* was detected as the dominant fungus with the symptoms reported (Fig. 1). On common bean, we detected *P. griseola, U. appendiculatus, F. solani, F. oxysporum, and C. lindemuthianum* symptoms present on the leaves. The dominant foliar disease was caused by *P. griseola* (Fig. 1d, e).

### Species differences in responses to Ri, Ta, and Ri × Ta inoculations

**Fig. 2:**
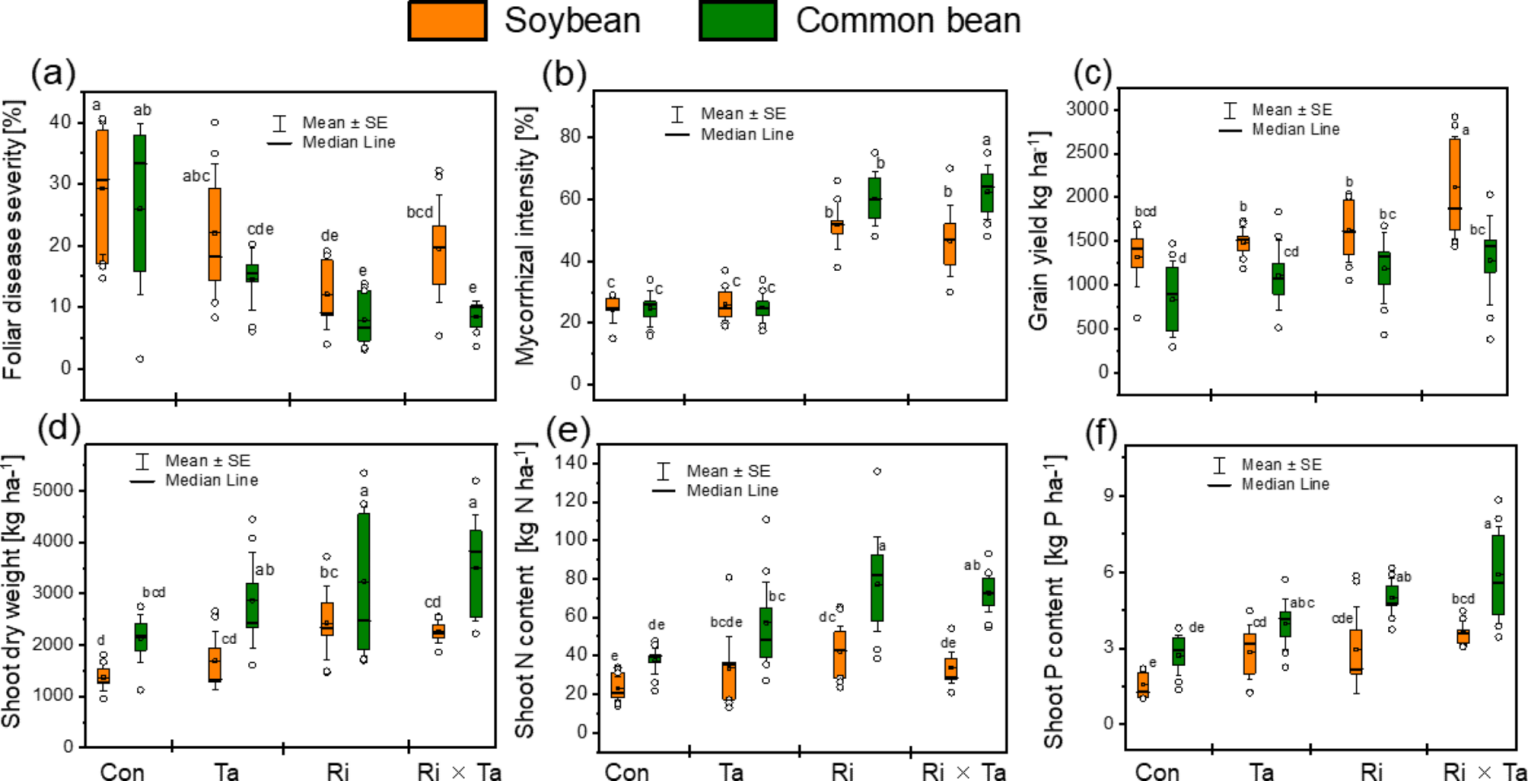
Foliar disease symptoms (**a**), mycorrhizal colonization intensity (**b**), yield (**c**) shoot dry weight (**d**), shoot N (**e**) and phosphorus (**f**) of the control (Con), *Rhizophagus intraradices* (Ri), *Trichoderma asperellum* (Ta), and the co-inoculated with Ri × Ta of the soybean and common bean plants grown under the humid forest conditions of Cameroon

Foliar disease symptoms on leaves, mycorrhizal colonization intensity, yield, shoot dry weight, N and P contents, and low-weight molecule synthesis of soybean and common bean plants of the control, Ri, Ta, and Ri × Ta treatments are reported in Fig. 2. In general, the common bean plants showed the lowest foliar infection rate, but a higher increase in the root colonization intensity, shoot dry weight and N and P contents than soybean when inoculated with Ri and Ta treatments. A two-way ANOVA revealed that the leaf yellowing and lesion symptoms, yield, shoot dry weight, and shoot N and P contents were significantly affected by the legume species and the microbes (Table 1; Fig. 2). Leaf yellowish and lesion symptoms were greater for the control treatments and reduced significantly in the Ri, Ta, and Ri × Ta treatments for both grain legumes (Fig. 2a). We also observed that mycorrhizal colonization was greatly altered due to inoculated microbes and the Ri and Ri × Ta treatments displayed the highest colonization rate on the common bean plants (Fig. 2b). Grain yield of common bean was lower compared to that of soybean plants for the different tested treatments (Fig. 2c). We observed a higher shoot dry matter, shoot-N and -P contents in common bean than in soybean for the control, Ta, Ri, and the Ri × Ta treatments (Fig. 2d, e, f). The interaction between legume species (S) and the fungal (F) inoculation (S ×F) was significant at *p* < 0.05 for the MCR only, implying that the grain legumes exhibited different behaviors to the root colonization, and common bean plants displayed higher colonization rate than soybean (Fig. 2b and Table 1).

**Table 1:** Analysis of variance (ANOVA) probabilities for the effects of leave lesion symptoms (LLS), mycorrhizal root colonisation (MCR), grain yield (YLD), shoot dry weight (SDW), shoot N (SNC), and Shoot phosphorus content (SPC) of Soybean and common bean tested under field condition of the humid forest zone of Cameroon

	DF	LLS		MCR		YLD		SDW		SNC		SPC	
		F	*p*	F	*p*	F	*p*	F	*p*	F	*p*	F	*p*
Legume specie (S)	1	10.1	0.002	0.98	0.3	20.3	< 0.001	30.6	< 0.001	41.7	< 0.001	17.2	< 0.001
Fungi inoculation (F)	3	13.8	< 0.001	47.1	< 0.001	6.4	< 0.001	6.4	0.008	8.7	< 0.001	9.1	< 0.001
S × F	3	0.75	0.5	4.4	0.007	1.1	0.3	0.94	0.4	1.6	0.3	0.66	0.57
N	72			72		72		72		72		72	
CV (%)		64		43.9		41.6		44.2		52.8		55.2	

### Genotypic or varietal differences in responses to Ri, Ta, and Ri × Ta inoculations

**Fig. 3:**
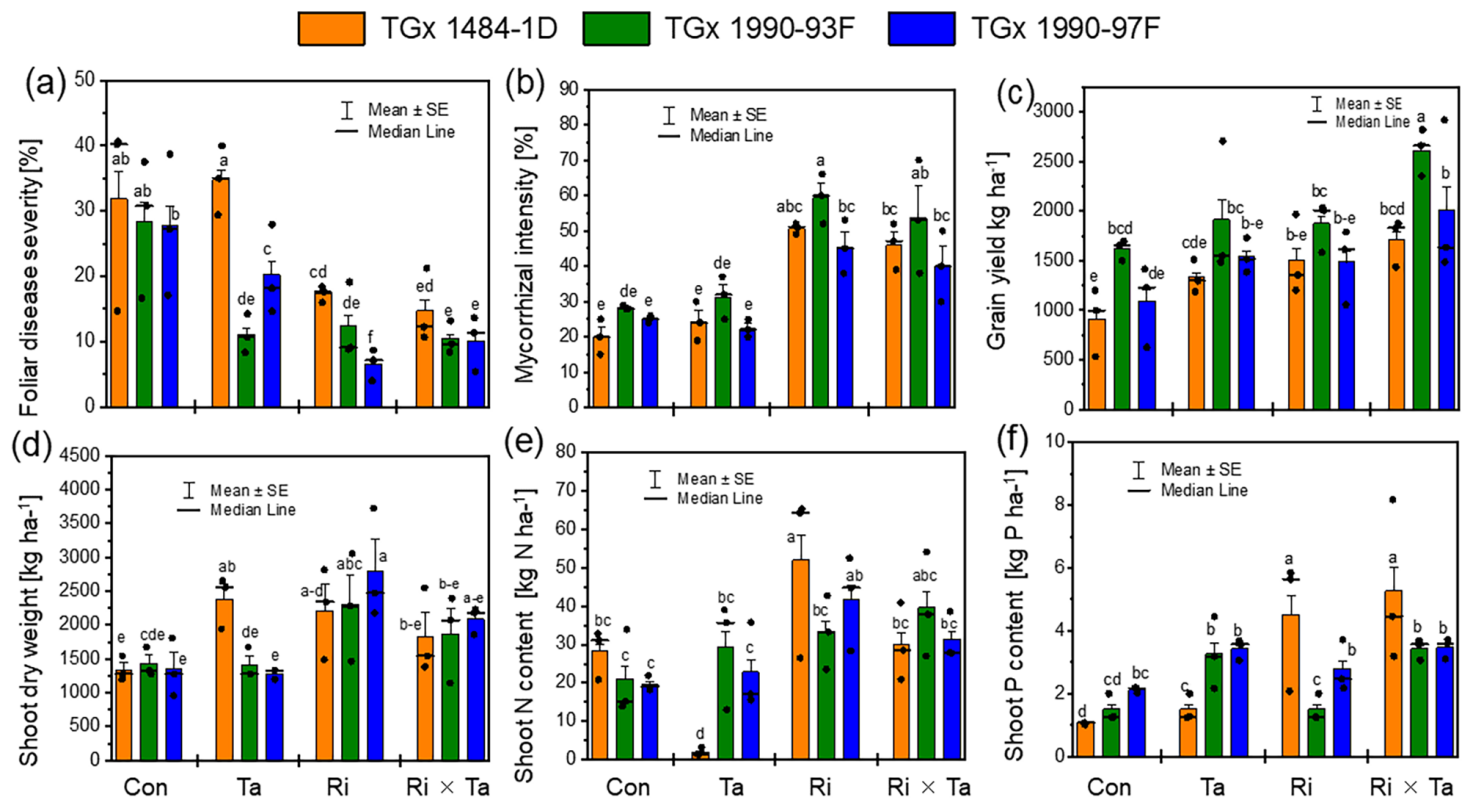
Foliar disease symptoms (**a**), mycorrhizal colonization intensity (**b**), yield (**c**) shoot dry weight (**d**), shoot N (**e**) and phosphorus (**f**) of the control (Con), *Rhizophagus intraradices* (Ri), *Trichoderma asperellum* (Ta), and the co-inoculated with Ri × Ta of the soybean genotypes grown under the humid forest conditions of Cameroon

The effects of genotype (G) and the Fungi inoculation on the foliar disease severity, mycorrhizal root colonization, and soybean grain yield were significant at *p* < 0.05 (Table 2, and Fig. 3). Among the soybean genotypes, lesion symptoms were lower for the genotype TGx 1990-97F inoculated with Ri, while lesions symptoms were higher in the control plants (Fig. 3a). The root colonization intensity of soybean plants inoculated with *R. intraradices* (Ri) and Ri × Ta was higher, compared to the control and the Ta treatments (Fig. 3b). The genotype TGx 1990-93F exhibited a higher grain yield compared other genotypes for the control Ta, Ri, and the Ri × Ta treatments, (Fig. 3c). There was a significant effect of the Fungi inoculation on the dry shoot weight. The soybean genotype TGx 1990-93F had higher root colonization intensity and a lower foliar disease infection, making it stronger and resistant to pathogens attack when co-inoculated with Ri × Ta fungi. Among the tested interactions between the genotype (G) and Fungi (F) inoculation, the G × F was significant regarding the shoot-P content, and the genotype TGx 1448-1D highly responded to the Ri and Ri × Ta inoculations than other treatments, indicating differently responded of soybean genotypes to fungi inoculation (Fig. 3; Table 2).

**Table 2:** Analysis of variance (ANOVA) probabilities for the effects of leave lesion symptoms (LLS), mycorrhizal root colonisation (MCR), grain yield (YLD), shoot dry weight (SDW), shoot N (SNC), and Shoot phosphorus content (SPC) of soybean genotypes tested under field condition of the humid forest area of Cameroon

		LLS		MCR		YLD		SDW		SNC		SPC	
	DF	F	p	F	p	F	p	F	p	F	p	F	p
Genotype (G)	2	9.1	< 0.001	6.6	0.005	8.1	0.002	0.4	0.66	2	0.15	0.05	0.94
Fungi inoculation (F)	3	8.5	< 0.001	34.9	< 0.001	7.7	< 0.001	6.9	0.002	2.9	0.05	6.2	0.002
G × F	6	1.8	0.14	0.5	0.8	0.24	0.9	0.24	0.9	0.93	0.5	4.4	0.003
N		36		36		36		36		36		36	
CV (%)		52.6		38.9		33.5		35.0		46.4		60.1	

**Fig. 4:**
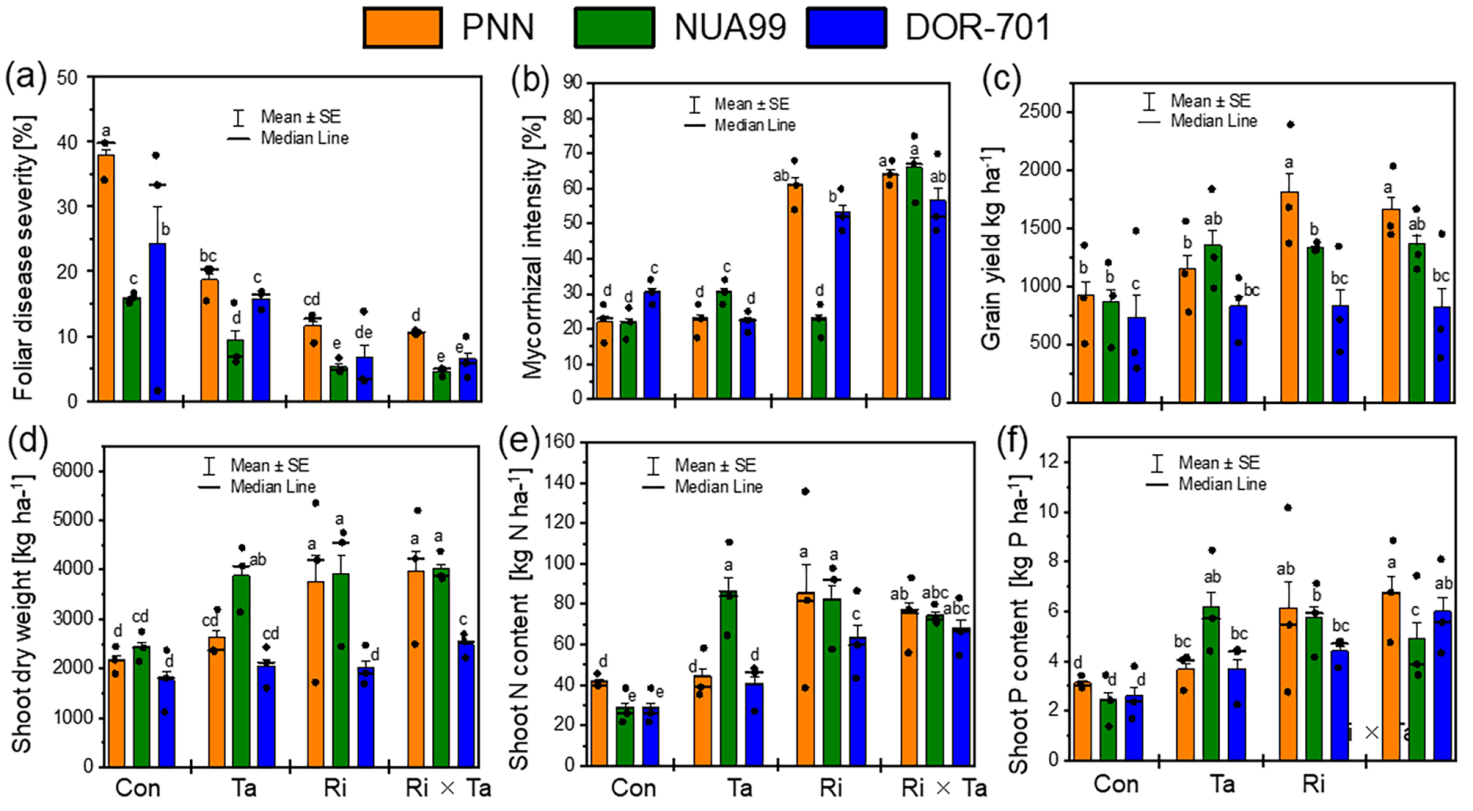
Foliar disease symptoms (**a**), mycorrhizal colonization intensity (**b**), yield (**c**) shoot dry weight (**d**), shoot N (**e**) and phosphorus (**f**) of the control (Con), *Rhizophagus intraradices* (Ri), *Trichoderma asperellum* (Ta), and the co-inoculated with Ri × Ta of the common bean varieties grown under the humid forest conditions of Cameroon

The probability values for the varietal (V) and the fungi effects were both significant at *p* < 0.05 for the leaves lesion symptoms, mycorrhizal root colonization, yield, and shoot dry weight of the tested common bean varieties (Table 3; Fig. 4). Beans plants inoculated with *R. intraradices* and the co-inoculation with Ri × Ta showed the lowest leave symptoms from fungi attacks but showed an increase in the root colonization, yield and shoot dry weight due to the Ri × Ta inoculation (Fig. 4a-d). The grain yield of the variety PNN inoculated with Ri was the highest compared to other imposed treatments (Fig. 4c). The Fisher value from the ANOVA revealed no difference between the varieties for the shoot-P and N contents of common bean plants, (Table 3), but the F inoculation effect was significant at *p* < 0.05 for the P and N contents (Table 3). We further observed that the interaction between bean varieties (V) and F inoculation was significant for the colonization intensity only, the bean varieties PNN, and DOR-701 always exhibited higher root colonization rates under Ri and Ri × Ta treatments, (Fig. 4b; Table 3).

**Table 3:** Analysis of variance (ANOVA) probabilities for the effects of leave lesion symptoms (LLS), mycorrhizal root colonisation (MCR), grain yield (YLD), shoot dry weight (SDW), shoot N (SNC), and Shoot phosphorus content (SPC) of common bean varieties tested under field condition of the humid forest area of Cameroon

		LLS		MCR		YLD		SDW		SNC		SPC	
	DF	F	*p*	F	*p*	F	*p*	F	*p*	F	*p*	F	*p*
Variety (V)	2	7.5	0.003	4.5	0.02	9.1	< 0.001	9.9	0.008	3.3	0.05	0.6	0.5
Fungi inoculation (F)	3	15.4	< 0.001	77.6	< 0.001	6.7	< 0.001	6.9	0.013	7.2	0.004	6.2	0.004
V × F	6	1.4	0.24	11.3	< 0.001	0.39	0.9	0.72	0.63	1	0.42	1.0	0.42
N		36		36		36		36		36		36	
CV (%)		73.7		48.3		43.6		38.2		42.6		44.8	

### Secondary metabolites accumulation in responses to Ri, Ta, and Ri × Ta inoculations

**Fig. 5:**
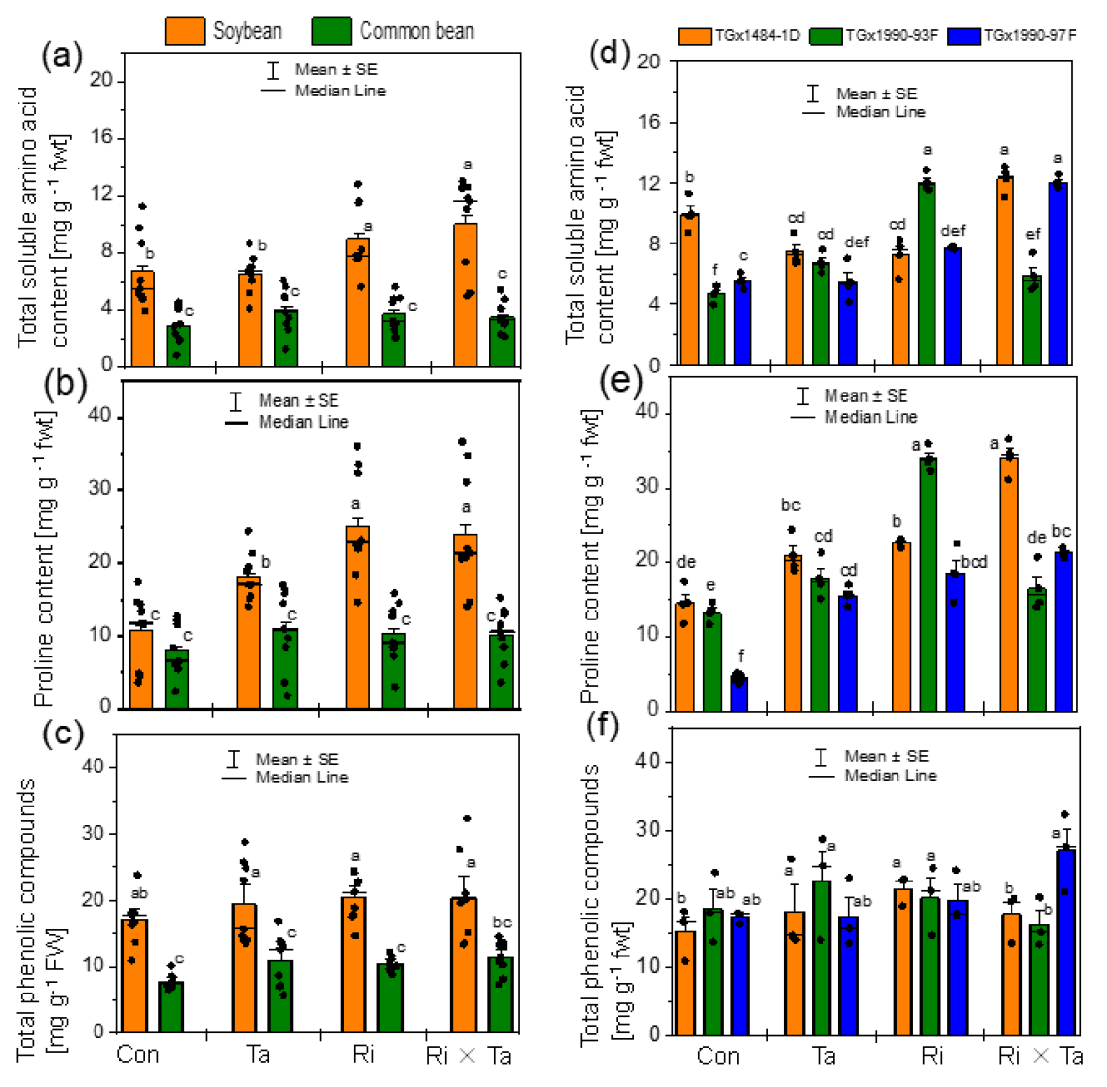
Total amino acid, proline, and total phenolic compounds in the leaves of soybean and common bean plants (**a, b, c**) and for the different soybean genotypes (**d, e, f**) of the control (Con), *Rhizophagus intraradices* (Ri), *Trichoderma asperellum* (Ta), and the co-inoculated with Ri × Ta treatments under the humid forest conditions of Cameroon

There was a significant effect of the F inoculation on the levels of amino acid, proline, and total phenolic compounds accumulation in the leaves of the studied grain legumes (Table S1). Highly significant differences between the grain legumes were observed, and the soybean plants accumulated higher amino acid, proline, and total phenolic compounds than the common bean in the different F inoculated treatments (Fig. 5a, b, c). The interaction between legume species (S) and the F inoculation (S ×F) was significant regarding the proline concentration (Table S2).

We further assessed the genotypic or varietal differences between the soybean genotypes (G) and common bean varieties for the amino acid, proline, and total phenolics compounds in the leaves. The two-way ANOVA revealed that the G and the F inoculation effects were highly significant (*p* < 0.001) for the amino acid and proline accumulations in the leaves of soybean plants (Table S2). The total amino acid concentrations in the leaves of soybean genotypes TGx 1484-1D, and TGx 1990-97F were higher for the Ri × Ta treated plants than others (Fig. 5d). *R intraradices* induced a higher amino acid content accumulation in the leaves of the soybean genotype TGx 1990-93F compared to other genotypes (Fig. 5d). About the proline content, we found that genotypes TGx 1484-1D inoculated with Ri, and TGx 1990-93F inoculated with both Ri and Ta accumulated the highest proline contents compared to other F inoculation treatments (Fig. 5e). There was no significant effect of the F inoculation and legume genotype on the total phenolic compounds in the soybean plants (Table S2; Fig. 5f). The G × F interaction effects was significant for the amino acid and proline accumulation in the soybean leaves (Table S3).

**Fig. 6:**
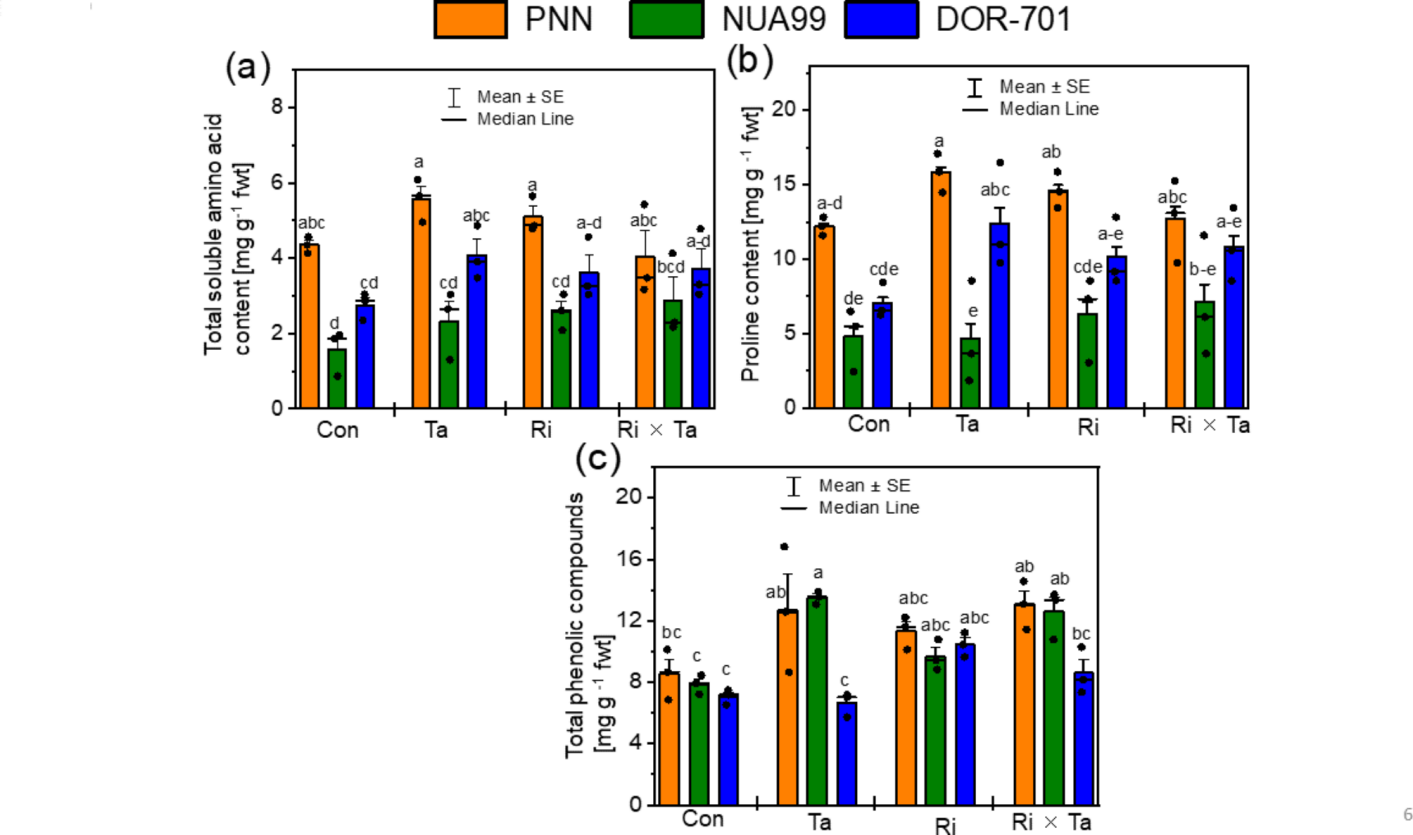
Total amino acid (**a**), proline (**b**), and total phenolic compounds (**c**) in the leaves of common bean of the control (Con), *Rhizophagus intraradices* (Ri), *Trichoderma asperellum* (Ta), and the co-inoculated with Ri × Ta treatments under the humid forest conditions of Cameroon

The total amino acid, proline, and total phenolic compounds in the leaves of common bean varieties are shown in Fig. 6. There was significant V and F effects on the total amino acid and total phenolic compounds of beans plants (Table S3). The PNN variety had higher total amino acid and proline accumulations in the Ta inoculated plants compared to others (Fig. 6a, b). Under the control, the PNN variety accumulated higher total amino acid and proline contents that significantly increased when inoculated with Ta fungi (Fig. 6a, b). The association of variety and the F inoculation significantly increased the total phenolic compounds in the leaves of bean plants, and the varieties PNN and NUA99 inoculated with Ta and Ri × Ta exhibited the highest contents compared to the rest of the treatments (Fig. 6c).

## Discussion

The exploration of genetic diversity among grain legume genotypes could allow to achieve a 30 to 50% yield increase in a properly managed production system. However, under the smallholder farmer production systems such as in the humid forest of Cameroon, various abiotic and biotic factors such as low nutrient availability of soil, pest and diseases curtail the success to increase yields [12, 24]. While extensive research work of the adverse effects of phosphorus deficiencies of soils to limit yield and crop production has been conducted, high pathogens variability among disease vectors isolate/strain in time and space limits the efforts towards breeding successful resistant cultivars. In the present study, we compared three soybean genotypes, and 3 common bean varieties inoculated with mycorrhizal, Trichoderma and a consortium of both fungi in terms of foliar diseases symptoms observations, root colonization intensity, yield, and accumulation of low molecules weight synthesis in the leaves. Our results demonstrated that common bean plants inoculated with Ri and Ta showed the lowest foliar infection rate and higher root colonization intensity, shoot dry weight, N and P contents than the soybean. The soybean genotype TGx 1990-93F showed the highest increase in the level of root colonization intensity and lower infection when co-inoculated with Ri × Ta fungi. The bean plants inoculated with *R. intraradices* and the co-inoculation with Ri × Ta showed the lowest leaf symptoms from fungi attacks but showed an increase in the root colonization, yield and shoot dry weight due to the Ri × Ta inoculation. Similarly, bean plants inoculated with *R. intraradices* and the co-inoculation with Ri × Ta showed the lowest symptoms of attacks, increasing root colonization, particularly for the PNN variety. The total amino acid and proline accumulation was higher for the soybean than beans plants in the different F inoculation treatments, and the soybean genotypes accumulated the highest contents of amino acid and proline in the control (10.1 mg g^− 1^ fwt) that significantly increased in the Ri × Ta treatment (13.4 mg g^− 1^ fwt).

### Interspecies variation for resistance against *soil-borne* fungi pathogens

We found significant differences between the tested grain legume species for the leave infection symptoms, mycorrhizal inoculation intensity, grain yield, shoot dry matter, and shoot-N and-P contents in responses to inoculation with Ri, Ta, and Ri × Ta. The Ri × Ta, and common bean generally performed better than soybean (Table 1; Fig. 2). Eke et al. [12] also observed a shoot dry weight increase and growth promotion effect on inoculated *P. vulgaris* with *T. harzanium* strain that was isolated locally. Trichoderma spp. are fast rhizosphere colonizers, which help exclude invading pathogens when the biocontrol fungi are applied to seeds or root [5]. The growth stimulation is often reported as a consequence of the intrinsic rapid proliferation potential conferred of the intrinsic rapid proliferation potential conferred to *T. harzanium* in the rhizosphere that outcompetes with pathogens fungi and enhances crop yield through more nutrient uptakes [5, 12]. Other mechanisms postulated for antibiosis include Trichoderma's secondary metabolites or secreted enzymes that inhibit pathogen growth or germination and contribute to mycoparasitism competition [5, 11]. Trichoderma fungus increases the availability of nutrients with low solubilities, like phosphates phosphates and other micro-nutrients by unlocking the biosynthesis of phenolic compounds, such as flavonoids, that impact plant growth [9]. With soybean, we lack data for effective comparison for the humid area of Cameroon. On Cacao, Tchameni et al. [31] also reported that inoculation with AMF and *T. asperellum* alone improved the growth and P uptake of inoculated cacao compared to non-inoculated control plants. The fact that the co-inoculation of Ri × Ta altered the root colonization intensity and promoted the of both soybean and common bean varieties offers an avenue for breeding improvement towards the developing of disease resistance varieties using the biocontrol approach.

The shoot P content of the common bean was always higher than the soybean, irrespective of the inoculation treatments in the present study. These two crops are annual dicotyledonous and members of the Phaseoleae legumes. The observed difference in P uptake could be attributed to the crop difference in P uptake and use efficiency which is likely higher in bean than soybean plants. Soybean has a relatively low root length densities that could influence the P uptake and subsequently their immobilization into areal parts [32].

### Genetic differences in legume responses to Ri, Ta, and Ri × Ta treatments

The results of the present study showed that both soybean and bean genotypes/varieties exhibited differences in responses to the imposed treatments. Genotypes TGx 1990-93F of soybean and NUA 99 of beans showed a high tolerance to foliar disease attacks on the control plants that were reduced in the Ta, Ri, or Ri × Ta treatments compared to other tested varieties. These two varieties offer offer a promising avenue in developing tropic grain legumes with high resistance to soil-borne fungal disease. In general, root and foot diseases severely impede grain legume cultivation worldwide, and breeding lines with resistance against individual pathogens have been developed for many grain legumes, including common bean, pea, and lupine [26]. But these resistances are often overcome by the interaction of multiple pathogens in field situations, making breeding programs for developing germplasm that consider both simultaneous attacks of various pathogens and the interplay with beneficial microbes very challenging. with cultivars of grain legumes generally showing low resistance levels [29]. Resistant germplasm is regularly detected among landraces, gene bank accessions, or related species or subspecies and the NUA 99 is an Andean-released cultivar from CIAT and could have several genetic improvements for tolerance to diseases.

In the present study, there were no significant interactions between the soybean genotypes and the fungi inoculation in terms of mycorrhizal root colonization rate under field testing conditions of the humid Forest of Cameroun. This could be because the different soybean genotypes used in the trials are improved varieties developed from tropical glycine max lines with no genetic difference in term of root colonization with mycorrhizal. With bean varieties, a landrace variety (PNN), and two improved varieties (NUA-99, DOR-701) by the International Center for Tropical Agriculture (CIAT) were used. Although these varieties are less explored for their mycorrhizal colonization intensity, these differences could have contributed to the alteration in root colonization rate.

4.3. *Low molecule weight (amino acid and proline contents) as mechanisms for Ta and Ri promotion of legume growth*.

Secondary metabolites are manufactured by plants, helping them to compete with environmentally exposed stresses. We analyzed three important secondary metabolites in particular the amino acid, proline, and phenolic compounds in the present study. Our results showed that soybean leaves accumulated higher acid amino, proline, and phenolic compounds than common bean plants that were significantly altered by the F inoculation (Figs. 5 and 6). The result suggests that *T. asperellum* effectively suppressed the soil borne pathogens, suppressed the soil-borne pathogens by activating several mechanisms such as competition, antibiosis, and induced plant defense systems (amino acid and proline, phenylalanine ammonia-lyase, and polyphenol oxidase activities) to combat and eliminate the pathogens. AMF facilitated the uptake of nutrients, in particular P, that helped the plant grow and achieve higher yield. Combining the Ri and Ta fungi, we recorded around 60% and 53% yield increases for soybean and common bean respectively compared to the control treatment. Under the greenhouse condition of Cameroon [12], there was an increase in bean growth due to co-inoculation with *T. harzanium* and *R. intraradices*. Plants accumulate phenolic compounds in their tissues as an adaptive response to adverse environmental conditions, and these phenolic compounds play a vital role in regulating various environmental stresses. The Ri alone and the combined Ri × Ta fungi inoculations increased the amino acid and proline contents in the leaves of soybean plants. At the same time, common beans inoculated with sole Ta also exhibited higher phenolic accumulations. Species preferences for secondary metabolite accumulation either inoculated with mycorrhizal, Trichoderma, or both microbes are less studied [16]. Rashidi et al. [16], when comparing three medicinal plants inoculated with AMF for their phenol concentrations in fruits and root organs, observed that *Digitaria sanguinalis* accumulated higher levels of phenols than *Solanum nigrum*, and *Ipomea purpura*. The results suggest Ri and Ta inoculation on common bean protected them from pathogen attacks. Possible gaps in the present work could be that we could have analyzed the various other types of secondary metabolites produced. The advances in analytic instruments such as the GC-MS, the entire metabolite profiles and the roles of volatile compounds will deserve future studies.

The interaction effects between the bean varieties and fungi inoculation were insignificant in terms of total amino-acid and proline accumulations, implying that the used fungi could not alter the leaf production. Secondary metabolite biosynthesis is differentially regulated in leaf tissues during growth and development. Until now, little was known about the expression patterns of genes involved in secondary metabolic pathways or their regulatory mechanisms. Using proteomic techniques to investigate the mechanisms of crosstalk between the common bean varieties and bacteria colonization [33]. Salavata et al. [33] found that the expression of 29 plant protein genes was upregulated, and the expression of 10 was downregulated. Upregulated proteins included those involved in protein destination/storage, energy production, and protein protein synthesis, whereas the downregulated proteins included those involved in metabolism, suggesting that the plant levels genetically control defense mechanisms associated with the induction of plant disease controls. The lack of interaction between common bean varieties and Fungi in the secondary metabolite’s synthesis calls for further research investigations to identify improved germplasms with enhanced secondary metabolites production for breeding efforts toward achieving new bean varieties adaptable to biotic or abiotic stressed conditions such as the humid Forest of Cameroon.

## Conclusion

We evaluated the effects of co-inoculation with mycorrhiza (*R. intraradices*) and Trichoderma (*T. asperellum*) fungi to colonize roots, reduce foliar disease severity promote growth, and accumulate secondary metabolites. We further investigated the inter-genetic variation among soybean and common bean co-inoculated with *R. intraradices*, and *T. asperellum.* We found that the soybean and common bean varieties exhibited differential genetic behaviors in response to the inoculation with mycorrhizal and Trichoderma treatments regarding amino acid and proline synthesis in the leave TGx 1484 1D was the best genotype when co-inoculated with mycorrhizas and Trichoderma. Common bean varieties PNN and NUA99 inoculated with *T. asperellum* alone or co-inoculated with *T. asperellum* and *R. intraradices* accumulated higher phenolic compounds that helped to improve resistance to pathogen attacks.

Research studies attempting to incorporate physiological markers like the production of acetic amino acids under the influence of mycorrhizal inoculation or combined inoculation with mycorrhizae and Trichoderma have not been successful in the humid forest zone of Cameroon. Our results offer a clue by proposing to the genetic improvement programs of the wetlands of Cameroon for the development of varieties tolerant to abiotic factors potential sources of genetic materials like the TGx 1990-93 F and TGx 1484 1D for soybean, PNN, and NUA99 for Common bean. These efforts will contribute to the development of tolerant germplasms aimed at increasing agricultural productivity. Further studies on low-molecular speciation and the nature of polyphenols will help to decipher the mechanisms of action linked to the introduced microorganisms.

### Electronic supplementary material

Below is the link to the electronic supplementary material.

Supplementary Material 1

## Data Availability

The datasets generated during and/or analyzed during the current study are available from the corresponding author on reasonable request.

## References

[1] Buenor AB, Kabiru MR, Bechtaoui N, Jibrin MJ, Asante M, Bouraqqadi A (2022). Grain legumes yields responses to rhizobia inoculants and phosphorus supplementation under Ghana soils: a meta-synthesis. Front Plant Sci.

[2] Herridge DF, Giller KE, Jensen ES, Peoples MB (2022). Quantifying country-to-global scale nitrogen fixation for grain legumes II. Coefficients, templates and estimates for soybean, groundnut, and pulses. Plant Soil.

[3] Peoples MB, Giller KE, Jensen ES, Herridge DF (2021). Quantifying country-to-global scale nitrogen fixation for grain legumes: I. Reliance on nitrogen fixation of soybean, groundnut, and pulses. Plant Soil.

[4] Siamabele B. The significance of soybean production in the face of changing climates in Africa. Moral MT, editor. Cogent Food Agric. 2021; 7:1933745. Doi: 23311932.2021.1933745.

[5] Mukherjee PK, Mendoza-Mendoza A, Zeilinger S, Horwitz BA (2022). Mycoparasitism as a mechanism of Trichoderma-mediated suppression of plant Diseases. Fungal Biol Rev.

[6] Nachilima C, Chigeza G, Chibanda M, Mushoriwa H, Diers BD, Murithi HM (2020). Evaluation of foliar Diseases for soybean entries in the pan-african trials in Malawi and Zambia. Plant Dis.

[7] Jemo M, Abaidoo RC, Nolte C, Horst WJ (2006). Genotypic variation for phosphorus uptake dinitrogen fixation in cowpea on low-phosphorus soils of southern Cameroon. J Plant Nutr Soil Sci.

[8] Liu J, Cai J, He R, Zhang X (2019). Influences of *Funneliformis mosseae* on the photosynthetic parameters and active secondary metabolites contents of *Astragalus membranaceus* and *Astragalus membranaceus* var. Mongholicus. ScienceAsia.

[9] Woo SL, Hermosa R, Lorito M, Monte E. Trichoderma: a multipurpose, plant-beneficial microorganism for eco-sustainable agriculture. Nat Rev Microbiol. 2022. s41579-022-00819-5. 10.1038/s41579-022-00819-5 36414835

[10] Ran Z, Ding W, Cao S, Fang L, Zhou J, Zhang Y. Arbuscular mycorrhizal fungi: Effects on secondary metabolite accumulation of traditional Chinese medicines. Wicke S, editor. Plant Biol. 2022; 24:932–8. 10.1111/plb.13449. 10.1111/plb.13449 35733285

[11] Zeilinger S, Gruber S, Bansal R, Mukherjee PK (2016). Secondary metabolism in Trichoderma – Chemistry meets genomics. Fungal Biol Rev.

[12] Eke P, Wakam LN, Fokou PVT, Ekounda TV, Sahu KP, Kamdem Wankeu TH (2019). Improved nutrient status and fusarium root rot mitigation with an inoculant of two biocontrol fungi in the common bean (Phaseolus vulgaris L). Rhizosphere.

[13] Abdelmoteleb A, Gonzalez-Mendoza D, Zayed O. Cell-free culture filtrate of *Trichoderma longibrachiatum* AD-1 as alternative approach to control *Fusarium solani* and induce defense response *Phaseolus vulgaris* L. plants. Rhizosphere.2022;100648. Doi: S2452219822001781.

[14] Balestrini R, Magurno F, Walker C, Lumini E, Bianciotto V (2010). Cohorts of arbuscular mycorrhizal fungi (AMF) in *Vitis vinifera*, a typical Mediterranean fruit crop: cohorts of AM fungi in vineyards. Environ Microbiol Rep.

[15] Yu L, Zhang Z, Zhou L (2022). Advances in the studies on symbiotic arbuscular mycorrhizal fungi of traditional Chinese medicinal plants. BIOCELL.

[16] Rashidi S, Yousefi AR, Pouryousef M, Goicoechea N (2022). Effect of arbuscular mycorrhizal fungi on the accumulation of secondary metabolites in roots and reproductive organs of Solanum nigrum, *Digitaria sanguinalis* and Ipomoea purpurea. Chem Biol Technol Agric.

[17] Sarkar AK, Sadhukhan S (2022). Unearthing the alteration in plant volatiles induced by mycorrhizal fungi: a shield against plant pathogens. Physiol Plant.

[18] Alizadeh S, Fallahi Gharagoz S, Pourakbar L, Siavash Moghaddam S, Jamalomidi M. Arbuscular mycorrhizal fungi alleviate salinity stress and alter phenolic compounds of Moldavian balm. Rhizosphere. 2021; 19:100417. Doi: S2452219821001130.

[19] Gbaporo FCG, Alain Heu SB, Mboussi et al. Patrice Zemko Ngatsi, William Norbert Tueguem Kuate, Sylvere Landry Lontsi Dida,. Performance of soybean genotypes (*Glycine Max* L.) against Asian rust (*Phakopsora Pachyrhizi* Syd.) in Cameroon. World J Adv Res Rev. 2021; 11:020–30.

[20] Djeugap F, Mefire M, Nguefack J, Ngueguim M, Fontem D (2014). Effet variétal et du traitement fongicide sur la sévérité de la maladie des taches angulaires et le rendement du haricot commun (*Phaseolus vulgaris* L.) à L’ouest-Cameroun. Int J Biol Chem Sci.

[21] Jemo M, Dhiba D, Hashem A, Abd-Allah EF, Alqarawi AA, Tran L-SP (2018). Mycorrhizal fungal community structure in tropical humid soils under fallow and cropping conditions. Sci Rep.

[22] Murphy J, Riley JP. A modified single solution method for the determination of phosphate in natural waters. Anal Chim Acta. 1962; 27:31–6. Doi: S0003267000884445.

[23] Heanes DL (1984). Determination of total organic-C in soils by an improved chromic acid digestion and spectrophotometric procedure. Commun Soil Sci Plant Anal.

[24] Novozamsky I, Houba VJG, van Eck R, van Vark W (1983). A novel digestion technique for multi-element plant analysis. Commun Soil Sci Plant Anal.

[25] Pastor-Corrales MA, Jara C, Singh SP (1998). Pathogenic variation in source of, breeding for resistance to *Phaeoisariopsis griseola* causing angular leaf spit in common bean. Euphytica.

[26] Phillips JM, Hayman DS. Improved procedures for clearing roots and staining parasitic and vesicular-arbuscular mycorrhizal fungi for rapid assessment of infection. Trans Br Mycol Soc. 1970; 55:158-IN18. Doi: /pii/S0007153670801103.

[27] Brundrett MC, Piché Y, Peterson RL. A new method for observing the morphology of vesicular–arbuscular mycorrhizae. Can J Bot. 1984; 62:2128–34. 10.1139/b84-290.

[28] McGonigle TP, Miller MH, Evans DG, Fairchild GL, Swan JA. A new method which gives an objective measure of colonization of roots by vesicular-arbuscular mycorrhizal fungi. New Phytol. 1990; 115:495–501. 10.1111/j.1469-8137. 1990.tb00476.x. 10.1111/j.1469-8137.1990.tb00476.x 33874272

[29] Yemm EW, Cocking EC, Ricketts RE (1955). The determination of amino acids with ninhydrin. Analyst.

[30] Olango N, Tusiime G, Mulumba JW, Nankya R, Fadda C, Jarvis DI (2017). Response of Ugandan common bean varieties to *Pseudocercospora griseola* and angular leaf spot Disease development in varietal mixtures. Int J Pest Manag.

[31] Tchameni SN, Ngonkeu MEL, Begoude BAD, Wakam Nana L, Fokom R, Owona AD et al. Effect of *Trichoderma asperellum* and arbuscular mycorrhizal fungi on cacao growth and resistance against black pod disease. Crop Prot. 2011; 30:1321–7. Doi: S0261219411001670.

[32] Henry A, Kleinman PJA, Lynch JP (2009). Phosphorus runoff from a phosphorus deficient soil under common bean (*Phaseolus vulgaris* L.) and soybean (*Glycine max* L.) genotypes with contrasting root architecture. Plant Soil.

[33] Salavati A, Taleei A, Akbar Shahnejat Bushehri A, Komatsu S (2012). Analysis of the Proteome of common bean (*Phaseolus vulgaris* L.) roots after Inoculation with *Rhizobium etli*. Protein Pept Lett.

