# Supplementary material for: Mycorrhizas and Trichoderma fungi increase the accumulation of secondary metabolites in grain legume leaves and suppress foliar diseases in field-grown conditions of the humid forest of Cameroon
Source: BMC Plant Biol. 2023 Nov 21;23:582. doi: 10.1186/s12870-023-04587-z (PMC10662906; doi:10.1186/s12870-023-04587-z)
Supplement: Supplementary file 1 — Supplementary Material 1 [file 12870_2023_4587_MOESM1_ESM.docx]

# Mycorrhizal and Trichoderma fungi inoculation increase secondary metabolites accumulation and suppress foliar diseases in field grown grain legumes: Increased yield and nutrient uptake implications’

Martin Jemo^1, *,^ S. Nkenmegne^2^, Alfred Balenor Buernor^1^, Anas Raklami^1^, Zachee Ambang^2^, Adamou Souleyamanou^2^, Yedir Ouhdouch ^1,3^ and Mohamed Hafidi ^1,3^

^1^ AgroBiosciences Program, College for Sustainable Agriculture and Environmental Sciences, Mohammed VI Polytechnic University (UM6P), Lot 660, Hay Moulay Rachid, 43150, Benguerir, Morocco

^2^ Department of Plant Biology, Faculty of Science, University of Yaoundé I, P.O. Box. 812, Cameroon.

^3^ Cadi Ayad University, Laboratory of Microbial Biotechnologies, Agrosciences and Environment, Faculty of Science Semlalia, P. O. Box: 2390/4000, Marrakesh, Morocco

Table S1. Soil chemical properties of soil analyzed in four replicates from the experimental sites at Nkolbisson, Yaounde Cameroun.

| Soil properties |  |
| --- | --- |
| pH (H_2_O) | 5.2 ± 0.2 |
| Soil available P | 3.3 ± 0.3 |
| Organic Carbon (g kg-^1^) | 129.6± 26 |
| N content (g kg-^1^) | 16.5 ± 4.5 g kg-^1^ |
| Silt content (%) | 10.2 ± 1.8 |
| Sand content (%) | 40 ± 5.3 |
| Clay content (%) | 48.9 ± 7.1 |
| Al^3+^ (cmol (+) kg^-1^) | 1.1 ± 0.5 |
| Ca (cmol (+) kg^-1^) | 1.1 ±0.1 |
| Mg (cmol (+) kg^-1^) | 0.33 ±0.01 |
| K (cmol (+) kg^-1^) | 0.08 ± 0.01 |
| Soil type (FAO classification) | Rhodic Kandiudult |

Table S2. Analyses of variance for total soluble amino acid (TAA), proline (PRO) and total phenols (TPH) contents of soybean and common bean varieties tested under field condition of the humid forest area of Cameroon.

|  |  | TAA | | PRO | | TPH | |
| --- | --- | --- | --- | --- | --- | --- | --- |
|  | DF | F | *p* | F | *p* | F | *p* |
| Specie (S) | 1 | 94.2 | <0.001 | 57.1 | <0.001 | 96.6 | <0.001 |
| Fungi inoculation (F) | 3 | 4 | 0.01 | 9 | <0.001 | 2.8 | 0.04 |
| S × F | 3 | 3.4 | 0.02 | 5 | 0.003 | 0.12 | 0.94 |
| N |  | 72 |  | 72 |  | 72 |  |
| CV (%) |  | 54.2 |  | 54.7 |  | 41.8 |  |

Table S3. Analyses of variance for total soluble amino acid (TAA), proline (PRO) and total phenols (TPH) contents of soybean and common bean varieties tested under field condition of the humid forest area of Cameroon.

|  |  |  |  |  |  |  |  |  |  |  |  |
| --- | --- | --- | --- | --- | --- | --- | --- | --- | --- | --- | --- |
|  | | DF |  | | | | | | | | |
|  |  |  | TAA | | | PRO | | | TPH | | |
|  |  |  | F | *p* | | F | *p* | | F | | *p* |
|  | | Soybean | | | | | | | | | |
| Genotype (G) | | 2 | 12.5 | <0.001 | | 33.4 | <0.001 | | 0.66 | | 0.52 |
| Fungi inoculation (F) | | 3 | 27.3 | <0.001 | | 363.3 | <0.001 | | 1.0 | | 0.4 |
| S × F | | 6 | 25.4 | <0.001 | | 18.9 | <0.001 | | 1.78 | | 0.14 |
| N | |  | 36 |  | | 36 |  | | 36 | |  |
| CV (%) | |  | 34.8 |  | | 42.8 |  | | 26.4 | |  |
|  | | Common bean | | | | | | | | | |
|  | | DF | TAA | | | PRO | | | TPH | | |
|  |  |  | F | | *p* | F | | *p* | F | *p* | |
| Variety (V) | | 2 | 30.5 | | <0.001 | 30.4 | | <0.001 | 13.9 | <0.001 | |
| Fungi inoculation (F) | | 3 | 3.6 | | 0.03 | 2.3 | | 0.1 | 9.0 | <0.001 | |
| v × F | | 6 | 1.1 | | 0.37 | 0.98 | | 0.45 | 3.6 | 0.01 | |
| N | |  | 36 | |  | 36 | |  | 36 |  | |
| CV (%) | |  | 36.7 | |  | 42.9 | |  | 26.4 |  | |
